# Patient experiences with technology enabled care across healthcare settings- a systematic review

**DOI:** 10.1186/s12913-020-05633-4

**Published:** 2020-08-24

**Authors:** Ann-Chatrin Linqvist Leonardsen, Camilla Hardeland, Ann Karin Helgesen, Vigdis A. Grøndahl

**Affiliations:** Department of Health and Welfare, Ostfold University College, Postal box code (PB) 700, NO-1757 Halden, Norway

**Keywords:** Digital health, Technology enabled care, Telehealth, Telemedicine, E-health, Digitalization, Patient experiences

## Abstract

**Background:**

Healthcare services are facing extensive challenges due to the increased proportion of elderly persons and persons with chronic disease. Technology enabled care (TEC) is a collective term for telecare, telehealth, telemedicine, mobile (m)-, digital- and electronic (e) health services. TEC is increasingly seen as a solution to many of the challenges facing the health sector. Patient perspectives may provide a useful evaluation tool for new healthcare technologies that have limited clinical data to support their effectiveness. More studies need to be done to better understand the acceptance of technology in healthcare. This review aim to summarize empirical studies exploring patient experiences with TEC. Findings in this study can be used to better understand what is needed to develop, implement and improve such services.

**Methods:**

Systematic searches were conducted in the Pubmed, Psycinfo, Cinahl, Embase, Cochrane systematic reviews and Cochrane clinical trials databases. These studies were systematically reviewed using the Preferred Reporting Items for Systematic Reviews and Meta-Analyses (PRISMA) guidelines, subjected to quality appraisals using the Critical Appraisal Skills Program (CASP), and synthesized via integrative analysis.

**Results:**

After removal of duplicates, languages other than English, and non-scientific records, 4087 titles and abstracts were screened. After assessment against inclusion and exclusion criteria, 69 records were screened in full-text, and underwent quality appraisal. 21 records were included in the integrative analysis. Patients’ experiences with TEC related to 1) technological features, namely functionality and appearance, and 2) evolving independence, namely empowerment, autonomy and security. Technological challenges lead to frustrations and negative experiences, while a stigmatizing appearance lead to patients not using the solution. Through the use of TECs, patients felt more empowered, learning about their condition, increasing awareness to their symptoms and treatment, and feeling more safe and self-efficient. Patient participation was seen as a central aspect of the development of the TECT, as well as when using it.

**Conclusion:**

This review deepens the understanding of patients’ experiences with technology enabled care solutions. Patients’ experiences not only relate to the practical/technical element of the device or solution, but to how this impact on their everyday life. Patient participation in development and planned use of such solutions should be considered an integral part in healthcare quality initiatives.

## Background

Healthcare services are facing extensive challenges due to the increased proportion of elderly persons and persons with chronic disease [1–3]. Despite increasing treatment complexity, hospital length of stay is decreasing [4]. In addition, there are not enough healthcare professionals to manage the increasingly complex patient care needs [5]. These societal changes challenge the structure, finances and capacity of all healthcare service levels. Technology enabled care (TEC) is a collective term for telecare, telehealth, telemedicine, mobile (m)-, digital- and electronic (e) health services. TEC involves the convergence of health systems, digital media and mobile devices, which enables healthcare professionals and patients to access data and information more easily [6]. It is increasingly seen as an integral part of the solution to many of the challenges facing the health sector.

Most people nowadays own a smartphone or a tablet. This enables for patient participation, e.g. through the use of mobile applications, or apps [7–9]. The development and utilization of commercial smartphone apps are extensive and increasing, also related to management of health-related issues [10]. In addition, solutions for remote patient monitoring, where patients outside conventional clinical settings have been monitored with help of technology, have been implemented with the aims of increasing access to care and decrease healthcare delivery costs. Nevertheless, results are inconclusive on whether such solutions have the desired effects [11–14]. For example, research on remote monitoring of patients with lung cancer indicated that patients felt well informed, but that they lacked preparation for allpossible problemsthey could experience [15]. A recent study found that when daily automated monitoring, self-management coaching and follow-ups using guideline-based decision support were combined with in between-visit care, there were significant reductions in symptom burden overall for cancer patients beginning chemotherapy [16].

At the same time, healthcare services are moving away from the “doctor-knows-best” approach, towards a focus on person-centeredness, and with increased levels of patient-participation [17, 18]. Research has shown that focus on person-centeredness leads to improved patient satisfaction, better health, a reduction in the number of hospitalization and re-hospitalizations, as well as economic benefits [19, 20]. This is why national and international organizations have emphasized the importance of including patients and their perspectives in the development and evaluation of healthcare services [21–24]. Patient perspectives can provide important, relevant insight into the nature of patients’ needs, the condition, and the treatment under consideration. Moreover, this may provide a useful evaluation tool for new healthcare technologies that have limited clinical data to support claims of effectiveness [25]. Experience-based measures differ from measures of satisfaction, which have previously been used as an index of how care has been received. ‘Satisfaction’ is often seen as the gap between patients’ expectations and actual experiences. Hence, ‘patients’ experiences’ provide more tangible information on how a service can be improved, and is less to prone to the influence of patient expectation, which is known to be influenced by varying factors [26–29].

There are various barriers to the deployment of TEC, such as concerns about quality, reliability, privacy and security. Moreover, it has been claimed that the design of TEC solutions have been technology-driven, without the involvement of the people they are made for [6]. A review from 2017 on recent advances in remote healthcare and patient monitoring claim that more studies needs to be done to better understand the acceptance of technology-based methods within the medical community and patients [30].

Consequently, this review aim to summarize empirical studies exploring patient experiences with technology enabled care solutions in relation to somatic diseases, treatment and care. Findings in this review may be used to better understand what is needed to develop, implement and improve such services.

## Methods

A systematic mixed studies review with an intergrated design was undertaken to integrate and synthesize findings from qualitative, quantitative and mixed methods studies [31]. The design was chosen to gain a broader knowledge of patients’ experiences with technology enabled care solutions in relation to somatic diseases, treatment and care, by exploring and describing studies that included different technology and different healthcare settings.

The literature was retrieved by searching in four electronic databases CINAHL via EBSCO, EMBASE and PsycINFO via OVID, PubMed via NCBI, in addition to the Cochrane systematic reviews and Cochrane clinical trials databases, in the period September 19th to October 20th 2019. A specialist librarian was consulted when developing the search strategy and also run the searches to ensure rigour in the search process. References were handled using the End-Note X8 and Rayyan QCRI software [32].

The Preferred Reporting Items for Systematic Reviews and Meta-Analyses (PRISMA) guidelines [33] were used to guide the systematic search and to structure the review, and the review adheres to the PRISMA guidelines (see supplement 1) .

The search strategies were developed based on the following PICO framework [34] (see Table 1). The searches were not limited to time of publication or to study design. Table 2 gives and overview of the search strategy in PubMed. Searches in the other databases were similar to the search strategy in PubMed, using the same terms and phrases, as well as Boolean operators.

**Table 1:** PICO: Digital Health Care –somatic health

Systematic review: patient experiences with technology enabled care			
P	I	C	O
Somatic health	Digitalization		Safety
Disease	Digital Health		Quality
Patient	Medical treatment		Patient participation
Illness	Health Care		Patient experience
	E-health		
	Technology enabled care		
	Telecare		
	Telemedicine		
	Telehealth		
	Computer based technologies

**Table 2:** Search strategy in PubMed

	PubMed Date for search: 19.09.2019	Hits
1	digitalization [tiab]	957
2	digitalisation [tiab]	178
3	“digital health” [tiab]	1235
4	telecare [tiab]	719
5	e-health [tiab]	2550
6	technology [tiab] enabled [tiab] AND care [tiab]	822
7	computer based technolog* [tiab]	133
8	telehealth [tiab]	4191
9	mobile technology [tiab]	1356
10	m-health OR mhealth [tiab]	4147
11	“mobile health” [tiab]	3581
12	telemedicine [ti] OR telemedicine [mesh]	26,786
13	1–12 med OR	37,279
14	experience* [ti]	239,920
15	perspective* [ti]	116,673
16	acceptance* [ti]	8655
17	participat* [ti]	35,793
18	preference* [ti]	27,473
19	14–18 med OR	424,513
20	patient [ti] OR patients [ti]	1,756,034
21	19 AND 20	42,745
22	Patient Satisfaction [majr]	32,722
23	Patient Acceptance of Health Care/psychology [majr]	14,173
24	21 OR 22 OR 23	84,518
25	13 AND 24	1044

### Eligibility criteria

Criteria for selecting studies were determined before the systematic literature search started, and was based on the aim of the review. The aim was explorative and descriptive, and the inclusion criteria reflect this.

Pre-defined inclusion criteria were:

- Patients aged > 18 years
- Patient experiences
- All healthcare settings (including but not limited to primary, intermediate, tertiary, home care)
- Somatic diseases/dysfunctions
- Scandinavian and English language
- Peer-reviewed

Exclusion criteria were:

- Conference abstracts
- Unpublished material
- Dissertations
- Review articles
- e-Health technologies, alerts and reminder systems and information resources (e.g. Internet)
- eElectronic health records

### Study selection

**Fig. 1:**
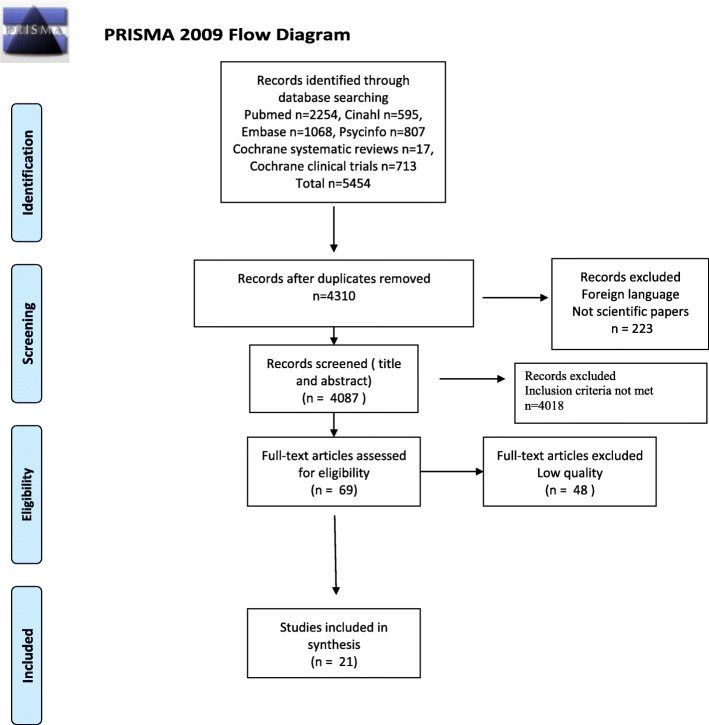
PRISMA 2009 Flow Diagram

A modified flow chart shows the identification and selection process (see Fig. 1). The electronic database searches identified 5454 records. Duplicates were removed which resulted in 4310 records, and a further 223 records were excluded based on language and not being peer reviewed. A total of 4087 records were divided in two halves. Two and two of the authors independently and blinded to each others, screened the titles and abstracts according to their relevance, to ensure that the eligibility criteria and the aim of the study were met. As a result, 4018 records were rejected. A total of 69 articles were assessed in full text by two and two of the authors to ensure that the inclusion criteria were met, which left 69 papers to be assessed for quality.

### Appraisal and data extraction

The Critical Appraisal Skills Programme Tools (CASP) for qualitative and quantitative studies [35] and a modified version by Nordström and Wilde-Larsson [36], were used for quality assessment of each paper included in the study. The appraisals related to e.g. whether the results were valid (clearly focused issue, appropriate method), if cases were recruited in an acceptable way, data collection justified, as well as ethical issues. The assessments were done by two authors independently. The results were then compared, and any differences on quality ratings were discussed until agreement was reached. A simple scoring system rated the papers to be of high, medium and low quality, and only high quality articles were included. A total of 48 articles were excluded based on low quality. All steps in the selection process, the appraisal and data extraction were performed by four independent researchers, that fulfills the requirements recommended by Higgins and Thomas [37]. Any differences or uncertainties were discussed by the authors until agreement was reached. In total 21 articles were included in the synthesis.

### Methods of synthesis

An integrative analysis inspired by Sandelowski et al. [31] was conducted to synthesize the data. This method gave data the possibility to be grouped by findings addressing the same phenomenon instead, of by methods used in the studies. Confirmation and refutation are exercised when seeking to establish convergent validation (or triangulation) between qualitative and quantitative studies, respectively. Confirmation occurs when the same finding (e.g. positive experiences with a TEC) is repeated within and across both qualitative and quantitative studies. Refutation occurs when a designated relationship yields divergent findings, or findings in direct opposition [31]. The findings might be seen as extending each other, and as a kind of transformation of findings to be able to combine them, which is essential in the integrative analysis.

## Results

### Study selection

In total 4310 records were identified, of which 4098 records were screened for title and abstract. Of these, 69 full text records were screened and quality assessed, leading to a total of 21 publications included in the review. The included studies represent research from the period 2008–2019, of which 20 was from the period 2013–2019. Of these, 12 studies used a qualitative design with interviews, six used a quantitative design with a survey, and three combined interviews and survey. The studies included a total of 695 participants (333 male), age range 18–88 years. Of the 21 studies, 19 reported the mean age of the participants. The mean of these were 61.9 years. The technology included telemonitoring (*n* = 5), sensors (*n* = 4), table computers (n = 4), fitbit (*n* = 3), applications (*n* = 2), a robotic rehabilitation device, a short-message self-management system, and an electronic pillbox. Six of the studies focused on type 2 diabetes, three on patients with chronic pulmonary lung disease (COPD), and two on cancer patients. Other conditions included Parkinsons’ disease, motor neuron disease, cardiac disease, stroke, hypertension, dialysis patients, as well as patients with chronic/persistent pain. The studies’ characteristics are presented in Table 3.

**Table 3:** Characteristics of articles included in the systematic review of studies exploring patient experiences with technology enabled care (*n* = 21), in alphabetic order

Authors/Country	Title	Aim	Design	Technology explored	Setting	Sample, including participants’ characteristics	Summary of relevant findings
Ando, Ashcroft-Kelso, Halhead, Chakrabarti, Young, Cousins, Angus 2019 United Kingdom (UK) [40]	Experience of telehealth in people with motor neurone disease using noninvasive ventilation.	To explore subjective experiences of telehealth as facilitated by the Careportal® device as a regular monitoring service amongst people with motor neuron disease (MND) who were using noninvasive ventilation (NIV).	Semi-structured interviews after completing a 24-week trial.	Telemonitoring system, using the Docobo^Ltd^Careportal® device. Allows for monitoring symptom changes, noninvasive ventilation (NIV) related issues, nocturnal blood oxygen saturation levels, and patient-ventilator interaction data. In addition a messaging system for patient-clinician communication.	At home	An opportunity sample of 7 patients with MND on NIV were recruited. Age: 49–71 years (mean 63 years) Gender: 2 women and 5 men Diagnosis: Amyotrophic lateral sclerosis	Five themes identified: - benefits of timely intervention - reducing the unnecessary - increased self-awareness - taking initiative - technical challenges
Cherry, Chumbler, Richards, Huff, Wu, Tilghman, Butler 2015 United States (US) [41]	Expanding stroke tele-rehabilitation services to rural veterans: a qualitative study on patient experiences using the robotic stroke therapy delivery and monitoring system programs.	To determine participants’ general impressions about the benefits and barriers of using robotic therapy device for in-home rehabilitation.	Qualitative in-depth semi-structured interviews after 3-months usage periode of 2 h of daily Robotic Assisted Therapy (RAT).	In-home robotic rehabilitation device: Individuals with residual upper limb impairments were given a Hand Mentor™device, while individuals with residual lower limb impairments were given the Foot Mentor™. The device is comprised a hand or foot peripheral component wired into a processing unit containing the pneumatic pump and a touchscreen interface. The device also has a cellular modem so that data from the therapy could be sent to a secure server to be monitored by a therapist. The device is powered through a wall-outlet.	At home	A convenient sample of 10 veterans who had experienced a unilateral ischemic or hemorrhagic stroke. Either hand function or foot function significantly limits activities of daily living Age: 52–88 years (mean 62 years) Gender: 10 men	Benefits of use: - increased mobility - a sense of control over therapy and scheduling - an outlet for physical and mental tension and anxiety - increased independence and mood improvement Barriers to use were few: - size and placement of the device - technical difficulties - wearing and adjusting the device
Fairbrother, Pinnock, Hanley, McClughan, Sheikh, Pagliari, McKinstry 2013 United Kingdom (UK) [42]	Exploring telemonitoring and self-management by patients with chronic obstructive pulmonary disease: a qualitative study embedded in a randomized controlled trial.	To explore patients and healthcare professional views on self-management in the context of telemonitoring in chronic obstructive pulmonary disease (COPD).	Semi-structured interviews six months into the randomized controlled trial.	Telemonitoring	At home	A purposively sample of 38 patients Age: 44–85 years (mean 67,4 years) Gender: 20 women and 18 men Diagnosis: COPD	Telemonitoring increased knowledge of their condition, reinforced their decisions to adjust to treatment or seek professional advice; and, through practitioner monitoring, provided a sense of reassurance. -Knowledge and empowerment -Accessibility and reassurance
Fisher, Hammerla, Rochester, Andras, Walker 2016 United Kingdom (UK) [43]	Body-worn sensors in Parkinson’s disease: evaluating their acceptability to patients.	To evaluate the acceptability of wrist-worn sensors in a Parkinsons disease population following assessment after both brief and prolonged periods of wearing.	Questionnaire with Lickert-style questions and free-text responses after use for 4 h in a research facility and after 1 week of continuously use at home.	Remote monitoring of symptoms in Parkinson’s disease PD) (using body-worn bilateral wrist-worn sensors.	Both in a research facility and at home	A sample of 34 patients Age: 50–86 years (mean 69 years) Gender not reported. Diagnosis: Parkinsons disease. Average duration of PD: 10 years (2–26 years)	94,1% were willing to wear the sensors at home, and 85,3% to wear it in public. -The sensors looks like it is well made -The sensor is comfortable to wear -The sensor does not feel heavy on my arm -The sensor is easy to take on and off Appearance: Physical properties could be improved. Wearing in public varied from not covering it up, to not like it to be questioned. Usability and Comfort were both positive and negative.
Georgsson, Staggers 2017 Sweden (Swe) [44]	Patients’ perceptions and experiences of a mHealth diabetes self-management system	To understand patients’ perceptions of (1) longer-term use of a specific mHealth system called Care4Life, (2) specific improvements needed for Care4Life, and (3) their needs for future diabetes self-management	Questionnaire and semistructured interviews after exit from a 6-month randomized controlled trial	Care4Life, an interactive short messaging service (SMS) diabetes self-management system for patients. Patients send in blood glucose, blood pressure, weight, exercises and medication adherence values, and receive reminders and advice about various aspects of their disease.	At home	A random sample of 10 patients participating in a larger RCT. Age: 50% were 60–69 years Gender: 5 women and 5 men Diagnosis: Type 2 Diabetes	Patients saw clear benefits in using the technology, and had favorable behavioral disease outcomes after using Care4Life. Attitudes: Positive overall system perception and general perception. Behavioral outcomes: Positive reminder, control and independence. Suggested improvements for behavioral support (highly individual): Information and disease related, and food and lifestyle related support.
Gorst, Coates, Armitage 2016 United Kingdom (UK) [39]	“It’s sort of a lifeline”: chronic obstructive pulmonary disease patients’ experiences of home telehealth	To explore the beliefs and perceptions of patients with chronic obstructive pulmonary disease (COPD) currently using home telehealth and who are not enrolled in a trial.	Semistructured interviews with patients who had been using telehealth from 6 months up to 3 years, with a mean of 17 months use.	Small-sized telehealth equipment allowing the patient to attach peripheral devices to take readings of their vital signs, with the individual readings displayed on the equipment. Larger sized telehealth equipment additionally allowed patients to view their data in graphs and charts.	At home	A sample of 8 patients as part of a larger study recruiting patients with COPD and/or Chronic heart failure. Age: 58–84 years (mean 68 years) Gender: 5 women and 3 men Diagnosis: COPD Average duration of COPD: 9 years (4–18 years)	Four superordinate themes: -perceiving benefits of “being watched over” as providing peace of mind -learning about the health condition and the impacts on self-management behaviour -active engagement in health service provision and better access to health care - valuing the importance of in-person care
Hanley, Ure, Pagliari, Sheikh, McKinstry 2013 United Kingdom (UK) [45]	Experiences of patients and professionals participating in the HITS home blood pressure (BP) telemonitoring trial: a qualitative study.	To qualitatively explore the experiences of patients and professionals taking part in a trial of BP telemonitoring based in a usual care setting, to identify what contributes to the effectiveness of the intervention, what limited its effectiveness and what may be required for success of the trial to be translated into routine care.	Semistructured interviews within a RCT of BP telemonitoring in routine care.	A validated electronic home blood pressure (BP) monitor and mobile phone technology that enabled the transfer of BP readings via SMS to secure website which was accessible to the user and their doctor and nurse, and also provided automated feedback to the patient. The BP monitor linked to a mobile phone wirelessly, via Bluetooth.	At home	A sample of 25 patients (20 from the intervention arm and 5 from control arm) Age: < 50 years: 5 50–59 years: 10 70+ years: 10 Gender: 10 women and 15 men Diagnosis: Hypertension	Patients became more engaged in the clinical management of their condition. The home monitoring system provided better evidence for action, facilitating rapid tailoring of medication. The role of telemetry-enabled home monitoring was motivating, an incentive to improve self-care and evidence which facilitated meaningful conversation and dialogue with professionals.
Jalil, Myers, Atkinson, Soden 2019 Australia (AU) [46]	Complementing a clinical trial with human-computer interaction: Patients’ user experiences with telehealth	To investigate how to discover patients’ user experiences in telehealth, eHealth, and mHealth in a clinical trial	Semi structured interviews and survey within a RCT.	A tablet computer, an automatic glucometer, and an automatic sphygmomanometer	At home	A sample of 9 patients Age: 52–74 years (mean 62 years) Gender: 4 women and 5 men Diagnosis: Type 2 diabetes mellitus for at least 12 months.	Two themes were identified: (1) the current design and how that fits with the patients’ needs -Lack of wireless capability -Undesirable experience from sphygmomanometer -Lack of visual data -Lack of medication name - Mismatch with life due to immobility of the device - Glucometer discomfort and pain (2) the patients experience of using the device depicted through their feelings and perceptions -Motivation -Build a habit -Awareness -Feel safe -Reduced doctor visits -Frustrations -Difficulty in measurement of blood pressure
Jamison, Mei, Ross 2018 USA (US) [47]	Longitudinal trial of a smartphone pain application for chronic pain patients: Predictors of compliance and satisfaction	To determine the long-term effects of using a smartphone pain app that allows chronic pain patients to assess, monitor, and communicate their condition to their physicians and that offers pain management strategies for users.	A longitudinal 6-month trial using a questionnaire at three and six months.	An app to assess, monitor and communicate patients’ status to their providers. Included an activity monitor and a Fitbit to track daily activity.	At home	A sample of 90 patients with chronic pain Age: 18–79 years (mean 47 years) Gender: 58 women and 32 men Diagnosis: Cancer and noncancer-related chronic pain for longer than six months	The app was easy to use. Those who used the app more often were more satisfied with the program. Satisfaction ratings diminished over time. Greater use of the app and frequent daily assessment entries were found to be related to an overall improvement in mood, but did not have a positive effect on pain or activity. Those who were more satisfied with the app reported more pain-related disability and were less active.
Kardas, Lewandowski, Bromuri 2016 Poland (PL) [38]	Type 2 diabetes patients benefit from the COMODITY12 mHealth system: Results of a randomised trial.	To assess patients’ assessment of their experience with COMMODITY12 system use within 6 weeks’ long clinical trial in DM2 patients- the “COMMODITY12” trial.	A 6 weeks RCT using a questionnaire at endpoint	COMMODITY12 system, composed of smart phone, and wireless connected sensors: -A Bluetooth-enabled glucometer, blood pressure reader and scale -A Bluetooth-enabled sensor of ECG, heart rhythm, and respiratory movements -A triaxial accelerometer – already built in the SmartHub (mobile phone) -MEMS™ - a patient adherence monitor, which has been used to assess patient adherence with oral antidiabetic agents they were using	At home	A sample of 60 patients (30 from the active arm and 30 from the control arm) Age: Mean 59 years Gender: 24 women and 36 men Diagnosis: Diabetes type 2 for at least six months prior to study	All telehealth system dimensions reached mean values of above four in a five-point scale, with maximum values for clearness of instructions, and ease of use (4.80 and 4.63 respectively), followed by general assessment, reliability of results and time spent on system use daily. Systems users asked for its strengths said system being fast, enabled them systematic self-monitoring and was easy to use. Weaknesses were frequent need to recharge and problems with glucometer strips.
Knudsen, Laustsen, Petersen, Hjortdal, Angel 2019 Denmark (DK) [48]	Experience of cardiac tele-rehabilitation: analysis of patient narratives	To explore patients’ experiences of tele-rehabilitation and the perceived gains of taking part in the program	Interviews after a 12-week hospital-based rehabilitation in an outpatient setting (phase II)	Monitoring equipment with a heart rate monitor with a sensor device, blood pressure monitor, weight scale, smartphone with built-in alarm and website. Information transferred from smartphone to a website. The patients and healthcare professionals had access to shared data on website.	At home	A sample of 7 patients. Age: 46–70 years (mean 58 years) Gender: 7 men Diagnosis: Ischemic heart disease or undergone heart valve surgery	Patients valued the cardiac tele-rehabilitation because it was not restricted to the hospital setting. Flexibility was assumed as an advantage. If activities were part of their daily lives, it lead to greater acknowledgement and commitment to the program. If not, the program was experienced as an extra challenge.
Lee, Greenfield, Pappas 2018 United Kingdom (UK) [49]	Patients’ perception of using telehealth for type 2 diabetes management: a phenomenological study	To explore patients’ perceptions of using telehealth for type 2 diabetes management	Semi-structured interviews with patients who had used telehealth from 1.5 years to 3.5 years.	Telehealth to monitor blood glucose, blood pressure and weight.	At home	A sample of 10 patients. Age: 49–77 years (mean 63 years) Gender: 8 women and 2 men Diagnosis: Type 2 diabetes for 4 to 33 years, mean time of 15.4 years.	Three themes for facilitating positive patient experience or acceptance of telehealth: Technology consideration -Initial perception of using technology for self-management -Telehealth usability concerns Service perceptions -Sense of security and comfort -Easy and convenience access to healthcare services -Privacy concerns -Continuity of care Empowerment -Patient education -Supporting self-care with telehealth system’s health trend analysis
Maglalang, Yoo, Ursua, Villanueva, Chesla, Bender 2017 USA (US) [50]	“I don’t have to explain, people understand”: acceptability and cultural relevance of a mobile health lifestyle intervention for Filipinos with Type 2 Diabetes	To access the acceptability and cultural relevance of the PilAm Go4Health program	A pilot randomized control trial using semi-structured post-program individual interviews after 3-months intervention and 3-months maintenance.	PilAm Go4Health program. A culturally adapted mobile weigth-loss lifestyle intervention including virtual social networking. Included using a Fitbit accelerometer, self-reporting of food/calorie intake and weight using the Fitbit diary app, and participate in a private Facebook group.	At home	A sample of 45 patients (22 from intervention group and 23 from an active wait-list control group). Age: Mean 58 years Gender: 28 women and 17 men Diagnosis: Non-insulin dependent Type 2 Diabetes	Four major themes were identified: -Culturally tailored support enhanced engagement -Mobile technology promoted personal agency -Progression from despair to self-efficacy -Further cultural tailoring addressing support mechanisms and improved site accessibility were suggested to improve intervention acceptability.
Maguire, Ream, Richardson, Connaghan, Johnston, Kotronoulas, Pedersen, McPhelim, Pattison, Smith, Webster, Taylor, Kearney 2015 United Kingdom (UK) [15]	Development of a novel remote patient monitoring system: the advanced symptom management system for radiotherapy to improve the symptom experience of patients with lung cancer receiving radiotherapy.	To (*a*) explore the feasibility and acceptability of the Advanced Symptom Management System in patients with lung cancer receiving radiotherapy and clinicians involved in their care and (*b*) assess changes in patient outcomes during implementation of the Advanced Symptom Management System with patients with lung cancer receiving radiotherapy in clinical practice	A repeated-measures, single-arm, mixed-methods study design involving poststudy semi-structured interviews and semis-structured questionnaires at baseline and end of treatment.	The Advanced Symptom Management System-R (ASyMS-R) enables real-time collection of PROM data as a mobile phone–based symptom monitoring system. Patients received self-care advice on their mobile phone or alerts were generated to a pager held by a health professional at the clinical site when symptoms were of clinical concern.	At home	A sample of 16 patients. Age: 42–85 years (mean 64 years) Gender: 11 women and 5 men Diagnosis: Lung cancer receiving radiotherapy	Only rarely did patients report problems in using the handset. They felt that the system covered all relevant symptoms, helped them manage the symptoms and effectively communicate with clinicians.
Minatodani, Chao, Berman 2013 USA (US) [51]	Home telehealth: facilitators, barriers, and impact of nurse support among high-risk dialysis patients.	To evaluate patients’ perceived effectiveness of and satisfaction with home telehealth self-monitoring and remote care nurse (RCN) support and to identify perceived facilitators and barriers encountered with RT use.	Mixed methods approach using semistructured interviews as part of a RCT	Remote technology to self-monitor the health (physiological measurements and answering 10 subjective health questions specific to end-stage renal disease and dialysis treatment) at home.	At home	A sample of 33 patients Age: 37–87 years (mean 60 years) Gender: 13 women and 20 men Diagnosis: End stage renal disease and dialysis treatment	Receiving efficient feedback from RCT. Were better able to identify changes in their health status. Experienced enhanced accountability, self-efficacy and motivation to make health behaviour changes. The most frequently cited barriers related to malfunctioning equipment or trouble with Internet connections, forgetfulness, and felling poorly.
Nordin, Michaelson, Eriksson, Gard 2017 Sweden (Swe) [52]	It’s about me: patients’ experiences of patient participation in the web behavior change program for activity in combination with multimodal pain rehabilitation	To explore patients’ experiences of patient participation in a Web Behavior Change Program for Activity (Web-BCPA) in combination with multimodal rehabilitation (MMR) in primary health care	Semistructured interviews with open-ended questions as part of a RCT.	Behavior Change Program for Activity in combination with multimodal rehabilitation (Web-BCPA). An eHealth solution for a biopsychosocial treatment of persistent musculoskeletal pain. The modules contained information, assignments, exercises, educational texts, videos and writing tasks.	At home	A consecutively sample of 19 patients at their 4-month follow-up of the RCT. Age: 27–60 years (Mean 45 years) Gender: 15 women and 4 men Diagnosis: Persistent musculoskeletal pain with a duration of at least three months in the back, neck, shoulder and/or generalized pain.	One theme: It’s about me with 4 categories: -take part in a flexible framework of own priority -acquire knowledge and insights - ways towards change - personal and environmental conditions influencing participation
Reeder, Demiris, Marek 2013 USA (US) [53]	Older adults’ satisfaction with a medication dispensing device in home care.	To examine the level of frail older adults’ satisfaction with medication dispensing device and assess perceived usefulness of the device by older adult home care patients.	A survey after 9 months as part of a prospective, longitudinal, three-arm RCT conducted over a one-year period	The MD.2 medication dispensing machine automatically dispenses pre-loaded medication and gives alerts to individuals about medication times.	At home	A sample of 96 patients Age: mean 80 years Gender: 63 women and 33 men Diagnoses: Chronic conditions present including diabetes, depression, COPD, dementia and heart disease.	Nearly all patients perceived the medication dispensing device as very easy to use, very reliable, helpful in the management of their medications, gave them peace of mind, and they would like to use the machine in the future.
Vatnøy, Thygesen, Dale 2017 Norway (NO) [54]	Telemedicine to support coping resources in home-living patients diagnosed with chronic obstructive pulmonary disease: patients’ experiences	To investigate how the patients experienced follow-up using a TM intervention, and the extent to which it supported and improved coping resources and independence.	Individual semi-structured interviews after a duration of 10 to 21 days of use.	The technological solution consisted of 1)a tablet with a video camera and pulse oximetry device for daily monitoring of pulse and oxygen saturation transmitted wirelessly to the tablet application, 2) software consisting of a questionnaire to measure subjective symptoms, 3) a questionnaire for symptom self-evaluation The follow-up included nurse with real-time follow-up video communication	At home	A convenient sample of 10 patients discharged to their homes after hospitalization. Age: 55–83 years (mean 65 years) Gender: 3 women and 7 men Diagnosis: COPD exacerbation	Two themes and 5 categories: The TM solution was experienced as comprehensible and manageable and provided meaning in daily life. -Handling and understanding the technology -Feelings related to technology use The TM solution intervention contributes to stress reduction caused by illness burden and facilitates living as normally as possible -Confidence and trust in the health service -Impact on independence and self-management -Integrity and meaning in life
Wall, Ward, Cartmill, Hill, Porceddu 2017 Australia (AU) [55]	Examining user perceptions of *SwallowIT*: a pilot study of a new telepractice application for delivering intensive swallowing therapy to head and neck cancer patients	To explore patients’ perceptions of *SwallowIT* and the delivery of preventive swallowing therapy during CRT via an asynchronous telepractice model.	A mixed methods approach using structured questionnaire at baseline and on completion of CRT and semi-structured phone –interviews at 3–12 months post-CRT.	An electronic app hosted on a secure external server and provided on a tablet. The system was designed to assist patients to complete independent home-practice of preventative swallowing exercises during CRT, and included instructional videos, images and text. Free text for communication between patient and speech pathologist.		A sample of 15 patients Age: 46–70 years (mean 59 years) Gender: 15 men Diagnosis: Oropharyngeal squamous cell carcinoma (SCC) planned for curative-intent chemo-radiotherapy	Patients felt comfortable, confident, motivated, supported, and that the method was effective. Few technical difficulties, and would not prefer face-to-face consultation in hospital. Themes identified were: -“It was really easy to use” (design, system inclusions, convenience) -“You’re motivated to do something” (extrinsic and intrinsic motivators) -“Difficult circumstances” (treatment side-effects, time constraints) –“You’re on the right track, but…” (Service delivery preferences, design preferences).
Welch, Balder, Zagarins 2015 United Kingdom (UK) [56]	Telehealth program for type 2 diabetes: usability, satisfaction, and clinical usefulness in an urban community health center.	To examine the usability, satisfaction, and clinical impact of a 3-month diabetes telehealth intervention for poorly controlled type 2 diabetes (T2D) patients.	A queationnaire at the 3-month follow-up	An electronic pillbox integrated into an existing diabetes remote home monitoring (RHM) device suite comprising a Blutooth®-enabled blood glucose meter and an automatic blood pressure monitor connected to a cellular hub for data uploaded to a clinical application. Telehealth nurse received regular RHM data alerts and called patients by phone at scheduled intervals.	At home	A sample of 29 patients Age: mean 61 years Gender: 17 women and 12 men Diagnosis: Diabetes type 2, not on multiple daily insulin therapy	High ratings of usability and program satisfaction from patients. Patients reported the home monitoring devices to be easy to use, easy to fit into daily routines and set up in a convenient place at home. The pillbox was in addition helping to organize medications, and easy to understand how to refill.
Woodend, Sherrard, Fraser, Stuewe, Cheung, Struther 2008 USA (US) [57]	Telehome monitoring in patients with cardiac disease who are at high risk of readmission.	To determine whether telehome monitoring of patients with cardiac disease at high risk of readmission would reduce hospital readmissions, improve functional status, and improve quality of life over usual care.	A randomized controlled trial using a questionnaire at 1 month, 3 months, and 1 year postdischarge for datacollection.	3 months of video conferencing with a nurse, daily transmission of weight and blood pressure, and periodic transmission of 12-lead electrocardiogram. Data were transmitted by telephone lines.	At home	A sample of 124 patients discharged from hospital Age: mean 66 years Gender: 75% men Diagnosis: 62 patients with heart failure and 62 with angina	Overall, the equipment was easy to use. Some difficulties with ECG and Video-conferencing. Relieved of worries. Increased confidence.

### Integrative analysis

Results from the integrative analysis of the 21 included papers show that patients’ experiences with technology enabled care solutions were divided into mainly two aspects; one aspect related to the technical features of the solution, and one aspect related to the solutions’ impact on the patients’ everyday life. The theme technical features included patients’ experiences with the practical use of a digital device or solution, both related to functionality and appearance of the solution. Moreover, patients experienced an evolving independence in their everyday life due to the technology, through an increased feeling of empowerment, autonomy and security.

### Technical features

To patients, it was imperative that the device or solution functioned well. For example, type 2 diabetes patients reported the importance of clearness of instructions, ease of use, convenience of location, and that the solution was easy to fit into daily routines. This implicated that they were satisfied with the solution [40, 47, 55, 56].

Nevertheless, patients also reported of technical challenges. One patient continuing stroke rehabilitation stated: «The keyboard is that frustrating I just couldn’t be bothered trying to get it to work because it wouldn’t» [40]. Other challenges reported by patients were oximetry transmission, device fault, mobile signal loss, immobility of the device, and difficulties placing the device on the body [40, 41, 46]. One study found that the device only functioned with wired internet that had to be connected through a cable through the telephone port in a patient’s house, which was reported as a problem by patients [46]. In another study, the modems reportedly took a long time to send the data and sometimes did not actually send the data at all, leading to patients not being able to use the solution [41]. In a study on stroke rehabilitation, patients reported of difficulty putting the device on and adjusting it by themselves [41]. A frequent need to recharge was also reported as a disadvantage [38]. Technical challenges lead to a feeling of frustration, and dissatisfaction with the device or solution [40, 46, 51].

Georgsson et al. found that type 2 diabetes patients saw clear benefits in using an m-Health system and had favorable behavioral disease outcomes after using it. Suggestions for improving the system were highly individual [44]. Need for individual tailoring was also reported in 57.8% of the respondents in a mobile weight-loss and lifestyle intervention for patients with type 2 diabetes, who reported that culturally tailoring of the program, addressing support mechanisms and improved site accessibility, enhanced their engagement [50].

Regarding appearance, 94.1% of patients with Parkinsons’ disease were willing to wear body worn sensors at home, while 85.3% were willing to wear it in public [43]. One patient stated that he “*Would prefer it to be a little smaller and with watch face as keep thinking it was a watch I was wearing”*. Another said that he would be “*Happy to wear (in public) but would not like members of public questioning what it is for as illness is private”.* A perceived stigma and embarrassment, affecting when participants chose to wear the device was also reported in another study [53]. For example, bulkiness of the monitor was reported a negative feature of the appearance of a digital solution [41].

### Evolving independence

Participants expressed an increased sense of independence from their newfound mobility for which they credited their use of the device [41, 58]. For example, 57.8% of the respondents in a mobile weight-loss and lifestyle intervention to patients with type 2 diabetes reported that the mobile health technology promoted their self-efficacy [50]. COPD patients reported that telemonitoring empowered self-management by enhancing their understanding of their illness, and providing additional justification for their decisions to adjust treatment or seek professional advice [42]. Moreover, a study on telemonitoring in motor neuron disease found that patients emphasized the benefits of timely intervention, reducing unnecessary actions and doctors’ visits, making patients more self-aware and allowing them to take initiative. Their acceptance of telemonitoring appeared to be a consequence of patients’ understanding of fluctuations in their physical well-being. Telemonitoring further enabled symptom awareness and interpretation of these symptoms [40]. Patients used words such as «motivation», «accountability», «habit», «comfort» and «awareness», indicating that patients felt more empowered through technology enabled care solutions [46, 49, 51, 55, 58, 59]. Empowerment also reduced frustrations related to technological features [58, 60].

COPD patients reported that the telehealth equipment lead to active engagement in health service provision and better access to healthcare [39]. Patient reports from an e-Health solution for a biopsychosocial treatment of persistent musculoskeletal pain showed that patients felt that the solution was «about me», and allowed them to take part in a flexible framework of own priority [52]. A study on telemonitoring in motor neuron disease found that patients emphasized the benefits of timely intervention, reducing unnecessary actions and doctors’ visits [40].

Several studies emphasized the importance of patient participation, understood as taking part in a structured and flexible concept with opportunities to influence and a variety of treatments to choose according to one’s own needs and priorities. A reasoning process between health care professionals, e.g. reading and documenting in the patient records was perceived as patient participation [39, 45, 52]. As an example, cardiac telerehabilitation was valued due to the flexibility, and that healthcare services were not restricted to the hospital setting. When activities were part of the patients’ daily lives, it lead to greater acknowledgement and commitment to the program. Otherwise, the program was experienced as an extra challenge [48].

Timely interventions were perceived as a result of regular monitoring of clinical information, contributing to both physical and psychological well-being. Participants appreciated their data being monitored by professionals who would make timely actions if they saw any irregular signs [40]. Technology enabled care solutions lead to a feeling of security, continuity of care, stress reduction, integrity, meaning and reassurance [41, 45, 49, 54, 57, 61]. For example, COPD patients perceived benefits of “being watched over” as providing peace of mind [39].

## Discussion

In this integrative review, 4098 journal articles were screened, and 21 articles were included which explored patients’ experiences with technology enabled care solutions. The integrative analysis showed that patients’ experiences were divided into two main aspects; technical features and evolving independence. Technical features was linked to functionality and appearance, while evolving independence was linked to empowerment, autonomy and security. Technology enabled care encompass a variety of instruments and modes of application. The interventions may be provided as an alternative to, in addition to, and/or alongside traditional healthcare services [62]. This integrative review did not limit to year, condition, disease (other than somatic), socio-demographics or study location. Hence, findings here give an insight into patient experiences with technology enabled care varying from telemonitoring to the use of applications, across age, gender, socio-cultural or geographic settings or diseases.

Even though we did not limit the search and inclusion criteria, all studies were conducted in patients receiving technology enabled treatment and/or care in their homes. Many countries struggle to stimulate digitalization and the adoption of digital services to improve health system performance, and to evaluate whether it actually improves health care. It is claimed that the results of digital transformation of health services will depend on the quality of the process, and the involved stakeholders [63].

The European Commision emphasize that further research is needed to evaluate digital health services’ potential to strengthen patient empowerment and provision of a basis for shared decision-making. Moreover, the Commision claim that there is insufficient data readily available to systematically assess the value of digital services [63]. This integrative review indicate that patients experience an evolving independence, due to empowerment, autonomy and security from using TEC.

Studies have documented feasibility and high patient adherence and satisfaction, but little evidence have been presented on impact on health outcomes [64, 65]. Findings in this integrative review show that studies focusing on ‘satisfaction’ most freequently use survey as method. In the studies included, most patients reported to be ‘satisfied’ with the technology. For example, one study showed that 67% of the participants (*n* = 124) were very satisfied, and 93% reported that they were willing to receive home telecare services in the future [57].

This integrative review shows that patient experiences increased empowerment through technology enabled care solutions. There is still no agreement regarding the elements that define patient empowerment. A recent review of the issue found 17 different definitions and described ten possible dimensions in empowerment [66]. Examples were patient participation in clinical decision-making, gaining control, motivation and knowledge acquisition [66]. A recent study highlight the perception of direct control on their treatment as the least valued element (2.87, SD 0.566) compared with care quality (3.75, SD 0.649) and relational support in the care context (3.91, SD 0.274) [67]. These dimensions coincide well with what patients experience as benefits of technology enabled care solutions.

In the studies included in this integrative review the mean age was 61.9 years. Moreover, age range was 18–88. In the US, 86% of adults aged 65 or older suffer from one or more chronic health conditions [68]. The concept of assisting the older adult through the use of technology so as to access healthcare services has been claimed to have enormous potential [69]. In one study, younger age was associated with greater technology use in health care, as well as capacity to engage in different aspects of health care activities [70]. Hence, high age may have impacted the findings in this study.

The successful implementation of innovative digital technology in healthcare services is a complex and time-consuming process. Digital transformation of the healthcare services requires advanced IT competence to be integrated directly into the provision of care and value co-creation with service users; healthcare personnel, patients and their relatives [71]. It has been claimed that e-Health implementation only leads to sustainable adoption when the implementation take into consideration, and aligns the e-Health content, with present contextual structures and the interventions in the implementation process [72]. For example, the study on digital medicine dispensers showed that healthcare professionals’ personal justification and rationale for such action is necessary, because their opinions and approval influence whether patients welcome this initiative or not [73].

Earlier studies focus on specific conditions, TECs, or healthcare levels. Findings in this study adds knowledge about patients’ experiences with a variety of TECs, across healthcare service levels and irrespective of patients’ condition. Since patient experiences’ provide more tangible information on how a service can be improved, and is less to prone to the influence of patient expectation [26–29], this information is important to include when developing and implementing TECs in the future.

## Strengths and limitations

The strength of this integrative review is that it is based on close collaboration with a specialized librarian who assisted in setting up search strategies, combinations and boolean operators. Moreover, screening of titles, abstracts and full-text of the records, quality appraisals, as well as the analysis, were conducted in close collaboration between the four authors. Any disagreements were discussed until consensus was reached. This process increases the validity and reliability of this systematic review.

The integrative review has several possible limitations. Firstly, we could have found more studies if we had searched in more databases. Nevertheless, the selected databases are the largest and most relevant for this specific research field and aim. Secondly, we limited our inclusion of articles to those of ‘high quality’, which may have excluded articles that could have added interesting information about patient experiences.

In addition, we could have included qualitative articles only, since qualitative approaches are more sufficient when aiming to explore experiences and perspectives [74]. Nevertheless, the quantitative studies mainly added information about positive and/or negative experiences, while the qualitative studies added in-depth information about challenges. Several of the studies used mixed-methods approaches, arguing the same. An integrative review method allows for a more comprehensive understanding of patient experiences with digital health solutions in a wide, somatic, healthcare approach [75].

One inclusion criteria was Scandinavian or English language. This may have excluded potentially useful studies, yet there is evidence that limiting studies in this way does not introduce significant bias [76]. We did not limit studies by year. This research area is evolving, and the number of publications has increased rapidly the last ten years. The first study in the literature search was from 1975, while the first included study undertaken quality appraisal and included was from 2008. The earliest studies focused on telehealth initiatives. By not limiting to year, we were able to explore studies in a wide, retrospective perspective, ensuring that we did not oversee any publications of interest.

In this integrative review we assess patients’ experiences. Of course, relatives’ and healthcare personnel’s experiences would have given more in-depth knowledge on different stakeholders’ experiences with specific technology or digital solutions, which also may impact patients’ experiences in total.

Scholars have debated whether research synthesis differences characterizing efforts to integrate qualitative research findings with the differences characterizing efforts to integrate quantitative research findings preclude mixed research synthesis [31]. Sandelowski et al. [31] claim that both qualitative and quantitative studies can be viewed as producing findings that can readily be transformed into each other. Aiming to explore patients’ experiences, we think that quantitative data as much as qualitative data adds to this knowledge.

## Conclusion

This integrative review deepens the understanding of patients’ experiences with technology enabled care solutions. Findings indicate that patients’ experiences not only relate to the practical or technical element of the device or solution, but to how this impact on their everyday life. Technology enabled care will probably be an imperative part of a comprehensive patient pathway in future healthcare services. Patient participation in development, implementation and utilization of such solutions should be considered an integral part in healthcare quality initiatives.

## Supplementary information

**Additional file 1.**

## Data Availability

Overview of excluded records, search strings and other background material of the current study are available from the corresponding author on reasonable request.

## Abbreviations

CASP: The Critical Appraisal Skills Program (CASP)
PRISMA: Preferred Reporting Items for Systematic Reviews and Meta-Analyses guidelines
TEC: Technology enabled care
